# The Wax "Problem."

**Published:** 1856-04

**Authors:** Christopher Johnston

**Affiliations:** Baltimore


### ARTICLE XII.

### *The Wax "Problem."*

At the close of an address delivered, March 4th, 1856, before the graduating class of the Baltimore College of Dental Surgery, Dr. Solyman Brown, of N. Y., proposed several "problems," and added that "peradventure their solution will be postponed for the exercise of the mental acumen of the twentieth century." A few hours study of the "first problem" has enabled me to spare the doctor the trouble of waiting so long.

"The first is this: Why does melted wax of the common honey bee, when allowed to cool slowly in a *thin* stratum, assume geometrical forms of the same average size and shape as the alveoli of the comb from which the wax is derived."

I pass over the forced analogy of the basaltic columns of Fingal's cave; and also the mysterious "connection with the original cells of the honey comb, and the instinct of the bee that forms them," for I find in the *wax itself* sufficient reason for the appearance above mentioned.

Beeswax is composed of two substances, *cerin* and *myricin*—the former, existing in the proportion of about 70 per cent. is brittle, *crystalline* and volatile—the latter is non-crystalline or amorphous.

If a quantity of wax be melted in any vessel whatever and then suffered to cool, the margin of the fused wax is first solidified, and presents a homogeneous appearance, because the more rapid cooling of the mass touching the vessel prevents the formation of any but minute crystals of *cerin*, which hold the congealed *myricin* in their meshes. Presently, however, as the temperature of the hotter middle portion is lowered, large crystals of *cerin* shoot out from the edges, or spring up elsewhere at the surface, and intersecting each other give rise to "geometrical figures," triangular, four sided, five and six sided, and of various sizes depending upon the *quantity* of wax employed, the thickness of the stratum and the gradual or sudden cooling of the substance. It is worthy of note that hexagons predominate, especially in square vessels, and that every where the angle of intersection tends most conspicuously towards 120°.

Hardly do the *cerin* crystals limit spaces at the surface than the enclosed *myricin* hardens and *nearly* obliterates the "figures," and so on until the surface becomes even: but if, when a *deep* vessel is used, we watch the moment at which the central figures become visible and then break the crust near the edge and *quickly* pour off the still melted wax, we will have upon the under surface of the crust permanent ridges intersecting each other.

It is an error to suppose that the surface of spontaneously cooled wax is uniform—on the contrary, the figures may still be observed by reflected light—and further, if the cake be again melted the *myricin* yields first to the heat, and the network of *cerin* crystals reappears there where fusion is beginning.

As I have remarked the thickness or thinness of the stratum does not affect the process further than by determining, in conjunction with the lesser or greater rapidity of cooling, the *size* of the crystals of *cerin*—indeed the whole matter may be readily observed by placing under the microscope a plate of heated glass, covered with a delicate varnish of wax and suffering it to become cold.

These facts are perfectly in accordance with *laws* which may be thus expressed: 1st, A *thin* stratum of a hot saturated solution of any crystallizable substance or the melted substance itself, as wax, sulphur, &c., will yield small crystals upon sudden cooling. 2d, A large mass of the saturated solution or of the melted body, will yield large crystals if the cooling process be conducted very slowly.

As to why *cerin* crystallizes and *myricin* does not crystallize, or in fact why wax exists at all, I doubt if the "mental acumen" of any century will ever be able to declare.

Christopher Johnston, M. D.

Baltimore, *March 6th*, 1856.

